# Genomic activation of the EGFR and HER2-neu genes in a significant proportion of invasive epithelial ovarian cancers

**DOI:** 10.1186/1471-2407-8-3

**Published:** 2008-01-08

**Authors:** Joanna Vermeij, Erik Teugels, Claire Bourgain, Ji Xiangming, Peter in 't Veld, Vanessa Ghislain, Bart Neyns, Jacques De Grève

**Affiliations:** 1 Laboratory for Molecular Oncology, Universitair Ziekenhuis, Vrije Universiteit Brussel, 1090 Brussels, Belgium; 2 Department of Pathology, Universitair Ziekenhuis, Vrije Universiteit Brussel, 1090 Brussels, Belgium; 3 Department of Medical Oncology Universitair Ziekenhuis, Vrije Universiteit Brussel, 1090 Brussels, Belgium; 4 Department of Internal Medicine and Medical Oncology, Ziekenhuis Netwerk Antwerpen (ZNA), 2170 Antwerpen, Belgium

## Abstract

**Background:**

The status of the EGFR and HER2-neu genes has not been fully defined in ovarian cancer. An integrated analysis of both genes could help define the proportion of patients that would potentially benefit from targeted therapies.

**Methods:**

We determined the tumour mutation status of the entire tyrosine kinase (TK) domain of the EGFR and HER2-neu genes in a cohort of 52 patients with invasive epithelial ovarian cancer as well as the gene copy number and protein expression of both genes in 31 of these patients by DGGE and direct sequecing, immunohistochemistry and Fluorescent in Situ Hybridisation (FISH).

**Results:**

The EGFR was expressed in 59% of the cases, with a 2+/3+ staining intensity in 38%. HER2-neu expression was found in 35%, with a 2/3+ staining in 18%. No mutations were found in exons 18–24 of the TK domains of EGFR and HER2-neu. High polysomy of the EGFR gene was observed in 13% of the invasive epthelial cancers and amplification of the HER2-neu gene was found in 10% and correlated with a high expression level by immunohistochemistry.

Mutations within the tyrosine kinase domain were not found in the entire TK domain of both genes, but have been found in very rare cases by others.

**Conclusion:**

Genomic alteration of the HER2-neu and EGFR genes is frequent (25%) in ovarian cancer. EGFR/HER2-neu targeted therapies should be investigated prospectively and specifically in that subset of patients.

## Background

Most ovarian cancers originate in the surface epithelium of the ovary and in particular from invaginations that are remnants of previous ovulation sites. The ErbB family of receptor tyrosine kinases plays a key role in normal ovarian follicle development and cell growth regulation of the ovarian surface epithelium [1].

Overexpression of EGFR (ErbB1) and HER2-neu (ErbB2) has been reported in ovarian cancer [2] and both receptors are commonly co-expressed [3]. In most studies overexpression of HER2-neu has been associated with a worse prognosis while no clear prognostic significance has been established for EGFR (ErbB1) expression [4-9].

Until now it has been unclear to what extent (over) expression of these genes reflects constitutional activation or merely reflects the physiological status of the normal progenitor cells or whether other mechanisms of contitutional activation exist in ovarian cancer.

The prognostic and predictive importance of these receptors and their downstream signalling pathways has been demonstrated in other malignancies and has led to the development of targeted therapies such as monoclonal antibodies (e.g cetuximab, panitunimab, trastuzumab) and small molecules tyrosine kinase receptor inhibitors (e.g gefitinib, erlotinib and more recently dual EGFR/HER2-neu inhibitors such as lapatinib) in lung cancer, colon cancer and breast cancer.

In breast cancer an increased copy number of HER2-neu defines a patient population that benefits significantly from treatment with trastuzumab and lapatinib.

In non-small cell lung cancer the presence of mutations in the tyrosine kinase domain of EGFR observed in a minority of patients with adenocarcinoma, is a critical determinant for tumour response to tyrosine kinase inhibitors [10-13]. Some studies also identify a high EGFR copy number and protein expression level as molecular predictors of tyrosine kinase efficacy in non-small cell lung cancer (NSCLC) patients [14-17]. For other malignancies such as glioblastomas and colorectal cancer, the predictive significance of molecular markers for benefit from anti-EGFR treatment with small molecule tyrosine kinase inhibitors or monoclonal antibodies is proposed but remains to be further defined [18-20].

These targeted therapies are also the subject of clinical trials evaluating their potential in gynecological maligancies.

In an unselected patient population with ovarian cancer and primary peritoneal carcinoma [7] only a modest effect (4% response rate) of gefitinib in an unselected patient population was observed suggesting the need to identify molecular markers that are predictive of response.

The EORTC is implementing a phase III clinical trial to examine the effect of adjuvant treatment wit erlotinib in unselected ovarian cancer patients in remission after first line chemotherapy (EORTC protocol 55041).

The aim of the current study was to examine the status of both the EGFR and HER2-neu genes with regard to the mutational status, gene copy number and expression level which could help to enrich for a patient population in which the benefit from targeted therapies could be electively examined.

## Methods

### Design of the study

The study was done retrospectively on archived ovarian tumour material. Data were compared to literature findings.

### Tissues

Archival ovarian tumour samples, collected from diagnostic or resection specimens of 52 patients were included in the analysis. The tissues were either fixed in Bouin and paraffin embedded or fresh-frozen blocks. The presence of adequate epithelial tumour tissue (80–100%) in each block and characterization of tumours was determined by a single pathologist (CB).

### Mutational analysis

From some patients biopsies were available from more than one disease site. Genomic DNA was thus extracted from 68 fresh tissue samples obtained from the 52 patients after macrodissection by the same pathologist (CB). Sections of the specimens used for DNA extraction contained a majority proportion of tumour cells. Tissue samples were first digested with proteinase K. DNA was further purified by phenol-chloroform extraction and sodium acetate-ethanol precipitation and subsequently redissolved in a Tris-EDTA buffer. Exons 18–24 encoding the tyrosine kinase domain of both EGFR and HER2-neu genes were amplified by two rounds of polymerase- chain-reaction assays with the use of Taq polymerase (Applied Biosystems). The PCR primers were designed with the help of the 'primer 3' program (Table 1 and 2).

**Table 1:** EGFR primers

	First step (5'—3')	Second step (5'—3')	Product Size(BP)
Exon18	F: agcatggtgagggctgag	F: *gctgaggtgacccttgtctc	258
	R: acagcttgcaaggactctgg	R: acagcttgcaaggactctgg	
Exon19	F: catgtggcaccatctcaca	F: catgtggcaccatctcaca	179
	R: ccacacagcaaagcagaaac	R: *ggtgtgtcgtttcgtctttg	
Exon20	F: cgaagccacactgacgtg	F: cgaagccacactgacgtg	244
	R: ctatcccaggagcgcagac	R: *ccgtatctcccttccctgat	
Exon21	F: cctcacagcagggtcttctc	F: cctcacagcagggtcttctc	215
	R: aatgctggctgacctaaagc	R: *ccgactggatttcg	
Exon22	F: tttttccaacagagggaaact	F: *cactgcctcatctctcacca	237
	R:aaagaaaatacttgcatgtcagagg	R:aaagaaaatacttgcatgtcagagg	
Exon23	F: ccactgccttcttttcttgc	F: *tttcttgcttcatcctctcag	205
	R: cagctaggcagtgtggacag	R: cagctaggcagtgtggacag	
Exon24	F: gcatcaccaatgccttcttt	F: *gcaatgccatctttatcatttc	200
	R: actcttcccaatggaagcac	R: actcttcccaatggaagcac	

EGFR primers used for amplification (hemi-nested PCRs).

* indicates that a GC clamp was added at the 5' end of the primer,

GC clamp: cgcccgccgcgccccgcgcccggcccgccgcccccgcccg

**Table 2:** HER2-neu primers

	First step(5'—3')	Second step(5'—3')	Product Size(BP)
Exon18	F: ccagcactgacccaccac	F: ccagcactgacccaccac	228
	R: ctcttgcccctcccatca	R: *agaactgccgaccacacc	
Exon19	F: cccacgctcttctcactcat	F: cccacgctcttctcactcat	183
	R: agagaccagagcccagacct	R: *gggtccttcctgtcctccta	
Exon20	F: tgtggtctcccataccctct	F: *ctctcagcgtacccttgtcc	230
	R: caaagagcccaggtgcatac	R: caaagagcccaggtgcatac	
Exon21	F: tacatgggtgcttcccattc	F: tacatgggtgcttcccattc	201
	R:catgggctagacaccactcc	R: *gctccttggtccttcaccta	
Exon22	F: tagcccatgggagaactctg	F: *ctccccacaacacacagttg	186
	R: agctctcatcctccctccag	R: agctctcatcctccctccag	
Exon23	F: actcctgaccctgtctctgc	F: actcctgaccctgtctctgc	220
	R: ctttcatgccccttgtgg	R: *aggacctcccaccctcct	
Exon24	F: accagactggagggggagt	F: *agaggcagcaagcacacag	203
	R: gagggtgctcttagccacag	R: gagggtgctcttagccacag	

HER2-neu primers used for amplification (hemi-nested PCRs)

* indicates that a GC clamp was added at the 5' end of the primer,

GC clamp: cgcccgccgcgccccgcgcccggcccgccgcccccgcccg

PCR products were submitted to Denaturing Gradient Gel Electrophoresis (DGGE), an electrophoretic separation method based on differences in melting behavior of double stranded DNA fragments that is recognized as very sensitive for the detection of mutations [21].

The power of this technique was evaluated and characterized in several aspects: (1) the hemi-nested PCR is very sensitive since the DNA equivalent of 3 cells was sufficient to generate an amplification signal (2) the DGGE can detect a hemizygous mutation even when the mutant cells used to prepare the DNA sample represented only 25% of the total cellular amount in dilution experiments, and (3) the 2 most prevalent somatic mutations found in the tyrosine kinase (TK) domain of EGFR (delE746-A750 in exon 19 and L858R in exon 21) in control samples (lung cancer cell lines NCI- H1650 and H255) were consistently detected with this technique. Corresponding white blood cell DNA samples of most patients were available as controls.

DNA fragments presenting an abnormal migration pattern on DGGE were submitted to complete nucleotide sequence analysis with the help of the DNA sequencing kit (Sequenase version 2.0, Amersham) followed by sequencing on gel (Life Technologies). Sequence data were analyzed visually.

### Immunohistochemistry and FISH analysis

Expression of EGFR (ErbB1) and HER2-neu (ErbB2) was determined by immunohistochemistry with the use of the EGFR PharmDx kit (Dako A/S, Glostrup, Denmark) and the Herceptest™ (Dako A/S). Four micrometer sections of each tumour block were cut. Tests were performed according to the instructions of each kit including the appropriate positive and negative controls. Both kits have been studied in normal ovarian tissue which stains negative for EGFR and HER2-neu.

Tumours were considered EGFR positive if a membranous staining intensity of ≥ 1+ was observed in more then 1% percent of the tumour cells (Dako EGFR pharmDX interpretation guide and [22].

HER2-neu was interpreted according to the Herceptest ™ criteria and scored as 1+, 2+, 3+. HER2-neu overexpression was defined as weakly (2+) or strong (3+) when complete membrane staining was observed in at least 10% of the tumour cells.

Fluorescence in situ hybridization (FISH) analysis was performed on cryosections using dual-color DNA FISH probes: for the determination of EGFR gene copy number a LSI EGFR (7p12) probe labeled with SpectrumOrange was used in combination with a CEP7 centromeric probe labeled with Spectrum Green (Abott Molecular, Des Plaines, Il, USA). For the detection of HER2-neu amplification a Pathvysion LSI Her-2 (17q11-12) probe labeled with Spectrum Orange was used in combination with a CEP 17 centromeric probe labeled with Spectrum Green (Abott). Thirty-four samples were available for combined FISH and immunochemistry analysis of EGFR.

EGFR copy number was scored as described by Cappuzzo *et al.*[14].

For HER2-neu only the cases with a 2+/3+ immunohistochemistry were submitted to FISH analysis (n = 6) based on a known correlation established in breast cancer and recent reports in ovarian cancer [9,23].

Amplification for both genes was defined as a ratio EGFR or HER2-neu/CEP 7 ≥ 2.

### Statistical analysis

Associations between factors were analyzed with a Fisher's exact test. A *p *value of 0.05 was taken as the limit for statistical significance.

## Results

### Tumour characteristics

Fifty-two invasive epithelial ovarian tumours including 27 serous cystadenocarcinoma, 4 mucineus cystadenocarcinoma and 21 endometroid carcinoma were characterized for the presence of mutations in the entire tyrosine kinase domain of both the EGFR and HER2-neu receptors using DGGE and sequence analysis. Of thirty-one invasive cancers, sections containing adequate amounts and quality of tumour were available to be examined for the expression of EGFR and HER2-neu and gene copy number by immunohistochemistry and FISH.

### Mutation analysis

DNA samples were extracted from fresh tumour material obtained from 52 patients. Previous studies done on DNA from distinct tumour sites on part of the cases in this series of ovarian cancers pointed to clonal heterogeneity within the malignant cell population with regard to loss of chromosome 11p [24]. Based on these observations it was decided to examine DNA samples representing different tumour sites when available. A total of 68 DNA samples were thus available for mutation analysis. The complete tyrosine kinase encoding domain was examined. No somatic mutations were detected in exons 18–24 of both receptors (Table 3). However, a variant DGGE migration pattern was frequently observed for exon 23 of the EGFR kinase domain. DNA sequencing revealed a point mutation at position 2709 C>T in this exon. This is a silent mutation (T903T) that does also not seem to influence mRNA splicing [25]. Among the 52 tumours investigated, 13 were heterozygous for this genetic variant, while 3 appeared to be homozygotes. Analysis of blood DNA samples available from 2 of these 3 patients could confirm the homozygosity. We also found this genetic variant in the germline DNA of 5 out of 20 (25%) control individuals. Therefore the T903T variant is to be considered a benign polymorfism frequently occuring in the Belgian population.

**Table 3:** EGFR and HER2-neu status of ovarian tumours

		EGFR (ErbB-1)						HER2-neu (ErbB-2)				
Histopathological subtype (N = 52)		IHC (N = 31)			FISH (N = 27)		TKD Mutation (N = 68)	IHC (N = 31)			FISH (N = 6) amplification	TKD Mutation (N = 68)
		0	1+	2/3+	< 2	P		0	1+	2/3+		
Adenocarcinoma:												

Serous	27	7	4	8	17	3	34	11	4	4	2	34
Mucinous	4	1	1	1	3	0	6	3	0	0	0	6
Endometroid	21	3	2	4	7	1	28	5	2	2	1	28

Fifty-two ovarian tumours were analyzed by immunohistochemistry, FISH and mutation status for EGFR (ErBb-1) and HER2-neu (ErBb-2). The IHC scores and definition of gene amplification are specified in the methods section. For EGFR no amplification was found. For HER2-neu the gene copy number was only examined in the 6 cancers with 2/3+ IHC.

N = number of cases, P = "high polysomy", 0 = absent, TKD = tyrosine kinase domain.

### Protein expression and gene copy number

**Figure 1:**
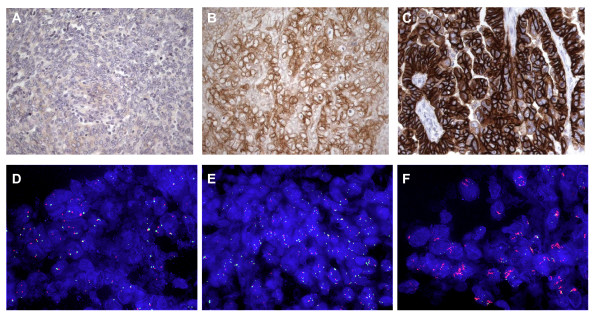
**Immunohistochemical and FISH analysis of EGFR and HER2-neu on ovarian tumours**. **A-C : Immunohistochemistry for EGFR and HER2-neu protein expression on paraffin sections from ovarian tumours (all 400× magnification)**. Examples of an EGFR score 0 (A), EGFR 3+ overexpression (B) and HER2-neu 3+ overexpression (C). **D-F : Fluorescence in situ hybridisation for the EGFR and HER2-neu gene on frozen sections from ovarian tumours (all 630× magnification)**. Examples of a tumour without EGFR amplification (D), a tumour showing polysomy 7 (E) and a tumour with HER2-neu amplification showing clusters of multiple gene copies (F).

EGFR immunostaining could be performed in adequately preserved samples from 31 patients. Positivity was found in 20/31 invasive epithelial cancers (64.5%) of which 13 (42 %) had an intensity of ≥ 2 (Table 3). FISH analysis for EGFR could be interpreted in 27 invasive epithelial cancers. No gene amplification was found in any of the samples. In 4 cases (13%) an increased number of EGFR copies (≥ 4 per nucleus) was detected in the presence of polysomy for chromosome 7. Such cases were defined as "high polysomy" as previously reported in NSCLC [14]. Immunohistochemistry on these polysomy cases yielded variable results one case being 0, one case 1+ and two cases 2+ for EGFR, giving no indication for a correlation with the gene copy number (Fig 1).

HER2-neu immunostaining was positive (≥ 2+) in 6 out of 31 invasive epithelial cancers cases investigated (19 %) of which three cases (10%) had an intensity of 3+ (Table 3). Six tumours had 1+ IHC positivity. Only the cases with ≥ 2+ immunohistochemistry were analyzed by FISH. In the 6 samples that showed either 2+ or 3+ positivity in immunohistochemistry HER2-neu gene amplification by FISH analysis was found in 3 cases (10%). One sample had a 2+ IHC score and 5 copies of the HER2-neu gene per nuclear section; two of the cases with a 3+ IHC score had > 15 copies of the HER2-neu gene per nuclear section (Table 4 and 5).

**Table 4:** EGFR and HER2- neu immunohistochemical expression

N = 31	EGFR				
HER2-neu		0	1+	2+	3+
	0	7	4	6	1
	1+	2	2	2	1
	2+	1	2	0	0
	3+	1	0	1	1

**Table 5:** EGFR and HER2-neu FISH results

	EGFR (N = 27)			
HER2-neu (N = 6)		≤ 2*	High polysomy §	Amplification**
	≤ 2*	20	4	0
	Amplification**	3	0	0

* Gene copy number, §as defined in [[14]].

** As defined in Methods.

One of the tumours with high level HER2-neu amplification also stained intensily (3+) for EGFR without increased gene copy number. One was EGFR 1+ and one EGFR negative.

The tumours (n = 4) displaying high polysomy for EGFR all stained negative for HER2-neu.

When the immunopositivity for EGFR and HER2-neu was compared (Table 4) no statistically significant correlation could be found between the expression levels An increased gene copy number for either of these two genes seems mutually exclusive (Table 5).

## Discussion

We have examined the concordant gene status (mutation status and copy number) and expression of both the HER2-neu and EGFR in a series of epithelial ovarian tumours. The mutational status of the genes could be examined in 52 invasive cancers. The IHC was evaluable in 31 and the FISH in 27 cancers.

Ovarian cancer has been examined in the past for some of these characteristics, but in separate reports. The current report is the first to examine the complete tyrosine coding domain of both receptors in a series of ovarian cancers. This analysis was complemented with the determination of gene copy number and immunohistochemical expression in samples in which this was possible, giving comprehensive information in a single series of ovarian cancers and for the two levels of relevance for prognostic or therapeutic applications of these genes: protein expression and gene status.

In breast cancer HER2-neu gene amplification is the major determinant for a potential benefit from trastuzumab treatment. In lung cancer studies [10,11,26] the majority of cancer associated somatic mutations occur in the tyrosine kinase domain of EGFR and Her-2 neu mainly in exon 18–21 and exon 19–20 respectively. Retrospective and prospective data in lung cancer indicate that an EGFR mutation is a stronger predictor for response and outcome under treatment with tyrosine kinase inhibitors than increased copy number [13,27,28].

In many studies in lung cancer, but also in other cancers, EGFR mutation analysis has been restricted to the exons known to be frequently mutated. This might be appropriate in a context of determining potential sensitivity towards small molecule tyrosine kinase inhibitors in a clinical setting of lung cancer. However, when examining other cancer types it remains to be determined whether other mutations might occur. Therefore, we examined the entire tyrosine kinase domain encoding exons (exons 18–24) of both EGFR and HER2-neu in a cohort of ovarian cancers. We have found no somatic mutations in 68 samples of 52 invasive epithelial cancers. Others have performed mutation analysis limited to the exons of the tyrosine kinase domain of EGFR known be mutated in lung cancer. Recently 2 activating mutations in the tyrosine kinase domain of EGFR (exon19 (delE746-A750), previously in NSCLC described have been reported in a series of 57 ovarian adenocarcinomas. In one patient this mutation correlated with an objective clinical response to treatment with gefitinib [7].

In another recent study [29] including mutation analysis of exon 18,19 or 21 of the EGFR TK domain in a series of 198 ovarian serous cystadenocarcinoma no mutation was found. This brings the total number of invasive epithelial ovarian cancer cases reported to 305 with a mutation rate of 0.7 % when only the exons known to be mutated in lung cancer are considered.

Activating and tyrosine kinase inhibitor (TKI) sensitizing mutations in the EGFR gene are thus very rare in ovarian cancer in contrast to adenocarcinoma of the lung in which the mutation rate varies between 5 – 45% depending on the phenotypic selection criteria and ethnic background of patients [14,17].

All of our patients were of Caucasian origin and it remains to be determined what the mutation rate would be in other ethnicities, for example Asian.

We did not study the extracellular domain of the EGFR receptor which is known to be mutated in glioma but also, albeit rarely in squamous cell lung cancer [30].

HER2-neu mutations have been described in a small subset (1.6 %) of lung cancers mainly in exon 19–20 [26,31]. These cancers and the patients in whom they occur have the same phenotype as the cancers in which EGFR mutations are found. The sigificance of these mutations with regard to sensitivity to HER2-neu inhibition remains undefined. One insertional mutation has been reported in exon 20 of the tyrosine kinase domain of HER2-neu in a series of 198 serous cystadenocarcinomas of the ovary [29]. Thus the cumulative data in 248 invasive epithelial ovarian cancers indicate a HER2-neu mutation rate of 0.4 %.

However the current report is again the only one that examined the complete TK domain. EGFR expression was observed in 64.5 % of invasive cancers, all adenocarcinomas, and in 13% percent of cases high polysomy as defined in lung cancer was found. This proportion of high polysomy is similar to that found by others [32] in primary ovarian cancers (9/64 or 14%). The cumulative data in 98 patients thus indicate a 13% rate of high polysomy of the EGFR gene in invasive epithelial ovarian cancer. The classification used in other work [29] is less clear and it is more uncertain what the potential biological significance of low levels of polysomy could be.

No correlation was observed between EGFR expression and EGFR gene copy number. This lack of correlation has also been observed by others [29] and in NSCLC [14].

HER2-neu overexpression was found in 19 % in our series, a proportion similar to other reports [33]. Three tumour samples had a 3+ staining on immunohistochemistry of which two tumours had a high level of gene amplification. A third sample with a 2+ IHC positivity had a lesser degree of gene amplification. This suggests that even with this sample size a clear correlation can be detected between expression level and gene copy number, as is also observed in breast cancer. The HER2- neu gene amplification rate is thus a non-negligible 10% in invasive ovarian cancers. There are currently no other data available in additional patient series. In breast cancer the gene status has the greater relevance with regard to prognostic and predictive implications. In breast cancer sometimes very high (double-digit) levels of gene amplification can be found in clinical samples, which has not been observed yet in ovarian cancer.

The expression status of the EGFR and the HER2-neu genes did not correlate although negativity for EGFR seems to associate with negativity for HER2-neu while vice-versa, negativity for HER2-neu can be associated with high EGFR expression, reflecting the overall lesser positivity rate for HER2-neu versus EGFR. Constitutional activation of the genes through increased copy number seems mutually exclusive.

What are the potential implications with regard to treatment with HER family inhibitors in epithelial ovarian cancer? Dramatic responses observed in lung cancer correlate strongly with the presence of EGFR tyrosine kinase domain mutations. The absence of these mutations in all but a very rare case of ovarian cancer probably will preclude such dramatic responses upon treatment of ovarian cancer with EGFR tyrosine kinase inhibitors. However, these very rare (< 1 %) patients eventually also should receive the opportunity to benefit from the exploration of TKI treatment, which would require the cost of performing a mutation analysis in a large number of patients only to identify rare cases, an effort that currently might not be considered as cost-effective.

When considering the overall data available it can be stated that the EGFR should be examined as a potential target for treatment in a significant minority of ovarium cancer patients and in particular for the 13% of patients with a cancer having high polysomy. In the BR21 study in NSCLC the strongest markers for survival benefit from treament with erlotinib was EGFR expression and high polysomy [13-17].

Clinical studies involving tyrosine kinase inhibitors in ovarian cancer are ongoing [34] and in the one study that has been published to date, analysis of EGFR gene copy number by FISH or CISH was not included [7]. The limited data available suggest a potential progression-free survival benefit. Four patients have been described with a progression -free survival of over 6 months (9 – 32 months) including one patient carrying an EGFR mutation [7].

If patients with high polysomy would benefit from anti-EGFR treatment and in particular TKI in analogy to lung cancer, and given the relatively low proportion of patients having such a high polysomy in their tumours, then it is to be feared that studies in unselected patient populations will dilute out this beneficial effect. Even if these studies are accompagnied by a retrospective analysis of these biomarkers, the results might suffer from insufficient power and biased sample analysis.

Therefore it is important to investigate the efficacy of EGFR inhibitors in (ovarian) cancer in patients prospectively selected for an altered gene status. In that context more uniform definitions of gene amplification and polysomy need to be established. Different study groups tend to use various definitions which makes a comparison of data difficult [14,29].

Monoclonal antibodies (eg cetuximab) directed against the extracellular EGFR domain do not seem to depend on the presence of mutations in the tyrosine kinase domain or gene amplification to induce responses in lung cancer [35]. Currently none of the aspects of EGFR status examined here has proven to be informative in predicting response to treatment with these monoclonal antibodies in other cancers [18-20,36].

Studies with these molecules have not been reported in ovarian cancer despite the high rate of immunohistochemical EGFR positivity.

HER2-neu is overexpressed in less than 1/5 of ovarian cancers. In patients selected for HER2-neu overexpression the overall clinical benefit of trastuzumab has been limited [37]. However, in breast cancer IHC expression is less relevant than the gene copy number as assessed by FISH or CISH.

If the analogy with breast cancer holds through, then HER2-neu directed treatment could benefit the ten percent of patients with invasive ovarian cancer identified as having a true gene amplification in their cancer (this study and [33]) and this should be explored prospectively again in this selected population, both as single agent and in combination with chemotherapy to investigate and exploit synergies as observed in breast cancer.

Separately the value of HER2-neu kinase inhibition should be evaluated in the very rare cases with a kinase domain mutation [29].

## Conclusion

We performed an integrated analysis of EGFR and HER2-neu protein expression and gene status in a series of epithelial ovarian cancers. Based on our findings and combined with data from the literature it can be concluded that EGFR/HER2-neu directed molecular treatments could benefit and should be investigated in one fourth of all invasive epithelial ovarian cancers.

The knowledge derived from retospective studies like this one should be used in the design of prospective trials investigating the value of EGFR/HER2-neu directed molecular treatments in epithelial ovarian cancer including the need for more uniform definitions of gene amplification and polysomy and selection of a patient population with a known altered gene status.

## Competing interests

The author(s) declare that they have no competing interests.

## Authors' contributions

JV and JDG carried out the design of the study and the analysis and interpretation of the data and drafted the manuscript. JX and ET carried out the mutation analysis. ET was also involved in the design of the study. CB is the pathologist responsable for the diagnosis, dissection of the tumour material and interpretation of the immunohistochemical staining. P'tV and VG performed the IHC and FISH analysis. BN participated in the draft of the manuscript.

All authors read and approved the final manuscript.

## Pre-publication history

The pre-publication history for this paper can be accessed here:


